# Interplay of Dirac electrons and magnetism in CaMnBi_2_ and SrMnBi_2_

**DOI:** 10.1038/ncomms13833

**Published:** 2016-12-16

**Authors:** Anmin Zhang, Changle Liu, Changjiang Yi, Guihua Zhao, Tian-long Xia, Jianting Ji, Youguo Shi, Rong Yu, Xiaoqun Wang, Changfeng Chen, Qingming Zhang

**Affiliations:** 1 Department of Physics, Beijing Key Laboratory of Opto-Electronic Functional Materials and Micro-nano Devices, Renmin University of China, Beijing 100872, China; 2 Beijing National Laboratory for Condensed Matter Physics, Institute of Physics, Chinese Academy of Sciences, Beijing 100190, China; 3 Collaborative Innovation Center of Advanced Microstructures, Nanjing 210093, China; 4 Department of Physics and Astronomy, Shanghai Jiao Tong University, Shanghai 200240, China; 5 Department of Physics and High Pressure Science and Engineering Center, University of Nevada, Las Vegas, Nevada 89154, USA

## Abstract

Dirac materials exhibit intriguing low-energy carrier dynamics that offer a fertile ground for novel physics discovery. Of particular interest is the interplay of Dirac carriers with other quantum phenomena such as magnetism. Here we report on a two-magnon Raman scattering study of AMnBi_2_ (A=Ca, Sr), a prototypical magnetic Dirac system comprising alternating Dirac carrier and magnetic layers. We present the first accurate determination of the exchange energies in these compounds and, by comparison with the reference compound BaMn_2_Bi_2_, we show that the Dirac carrier layers in AMnBi_2_ significantly enhance the exchange coupling between the magnetic layers, which in turn drives a charge-gap opening along the Dirac locus. Our findings break new grounds in unveiling the fundamental physics of magnetic Dirac materials, which offer a novel platform for probing a distinct type of spin–Fermion interaction. The results also hold great promise for applications in magnetic Dirac devices.

## Abstract (web summary)

The interplay between the low-energy carriers in Dirac materials and magnetism is likely to reveal many novel physical phenomena. Here, the authors use two-magnon Raman scattering to determine the exchange energies of two prototypical magnetic Dirac systems, CaMnBi_2_ and SrMnBi_2_.

Recent years have seen the emergence of a new class of materials whose low-energy carrier dynamics obey the Dirac equation, instead of the Schrodinger equation that describes most condensed matter systems. These so-called Dirac materials exhibit linear carrier dispersion and massless chiral excitations that give rise to novel quantum phenomena such as the ultra-high electron mobility and quantum Hall effect^1^,^2^,^3^,^4^. So far identified Dirac materials include graphene^2^,^3^, topological insulators^5^,^6^ and *d*-wave and iron-pnictide superconductors^7^,^8^.

One of the most intriguing aspects of Dirac materials is the interplay of their unique carrier dynamics with other quantum phenomena. A prominent case is magnetism, which may significantly change the electronic band structures of Dirac materials, as demonstrated by an antiferromagnetic (AF) long-range order in graphene observed in a recent experiment^9^. Further studies on the mutual influence of the Dirac-type electronic excitations and magnetism are impeded by the small size of available graphene samples (∼7 nm in width). A suitable model material system that possesses both Dirac carriers and magnetic order is essential to further exploration of novel physics and innovative device concepts in magnetic Dirac materials. The recent discovery of coexisting linear Dirac bands and long-range magnetic order in SrMnBi_2_ and CaMnBi_2_ provides an exciting platform for the study of magnetic Dirac materials.

Transport measurements reveal linear band dispersions near the Fermi energy in both AMnBi_2_ (A=Sr, Ca) compounds^10^,^11^,^12^,^13^. First-principles calculations indicate that such Dirac-type linear dispersions come from the 6*p*_*x*_ and 6*p*_*y*_ orbits of the Bi ions in the intercalated Ca(Sr)Bi layers, which are slightly hybridized with the *d* orbits of the Ca or Sr ions^10^,^12^,^14^,^15^. The calculated band structure has been verified by angle-resolved photoemission (ARPES) experiments^10^,^16^,^17^. The transport measurements also indicate an AF transition around 290 K^10^,^11^,^12^,^13^. The ground state predicted by first-principles calculations has a checkerboard AF order of Mn^2+^ spins, with a spin moment of ∼4 μ_B_^14^,^15^. Subsequent neutron diffraction measurements^18^ confirmed the AF transition and the estimated size of the magnetic moment, and the experiment also demonstrated that CaMnBi_2_ and SrMnBi_2_ have the C-type and G-type AF structures, respectively. Moreover, it is shown that magnetic ordering could open an energy gap in Dirac fermion band in CaMnBi_2_ but not in SrMnBi_2_ at the mean field level^18^. The AMnBi_2_ compounds comprise alternating Ca(Sr)Bi layers accommodating two-dimensional Dirac electrons and MnBi layers containing a long-range magnetic order and this configuration is similar to the case reported in the magnetic graphene. The availability of large-size AMnBi_2_ crystals allows experimental explorations of the interplay between the coexisting magnetic order and Dirac electrons.

In this work, we report on Raman-scattering measurements of two-magnon excitations in SrMnBi_2_ and CaMnBi_2_. From the measured and calculated Raman spectra, we have determined, for the first time, the nearest-neighbour (*J*_1_), next nearest-neighbour (*J*_2_) and interlayer (*J*_c_) exchange energy. By comparison with a reference system BaMn_2_Bi_2_, we find an unusual enhancement of the interlayer exchange couplings between neighbouring magnetic layers via the intervening Dirac carrier layers. We further examined the effect of the magnetism on the Dirac electron band structure using a spin–fermion model. Our results show that the enhanced interlayer exchange coupling drives a charge-gap opening along the Dirac locus in CaMnBi_2_. Unlike the effect of the spin–orbit coupling (SOC), the gap opened by the AF ordering allows both the upper and lower branches of the Dirac locus to cross the Fermi level, which well explains recent ARPES measurements^16^. The present study addresses a fundamental issue in magnetic Dirac materials, that is, the interplay between Dirac carriers and magnetism. The materials systems studied here provide a novel platform for probing a new type of charge–moment interaction where the magnetic and conducting layers are coupled but well separated, making these materials a new family of prototypes well described by the spin–Fermion model. This unusual separation of conducting (Dirac) charge and magnetic moments allows a delicate manipulation of the interaction between the charge and moment subsystems, which can be explored for innovative spintronic applications.

## Results

### Identification of two-magnon Raman spectra

**Figure 1:**
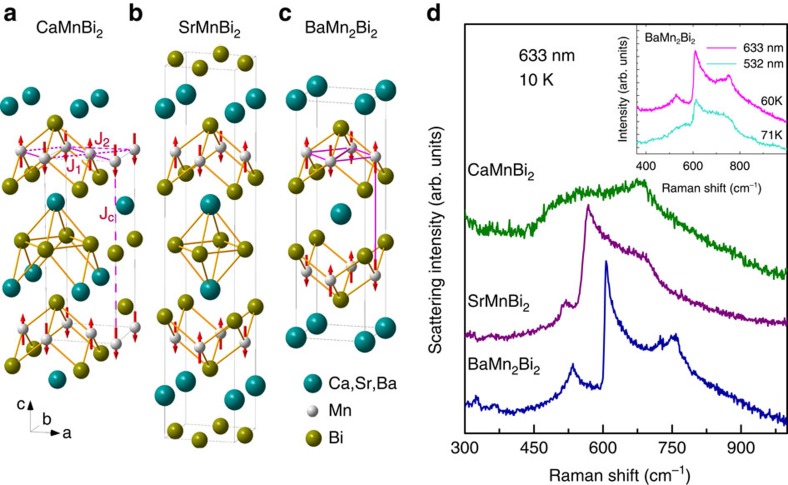
Crystal and magnetic structures and two-magnon Raman spectra. (**a**–**c**) The crystal and magnetic structures of the three compounds^18^,^19^. (**d**) Their two-magnon excitations measured at 10 K. The inset shows the two-magnon spectra at different excitation energies. The spectra were collected in an unpolarized configuration to obtain a better signal-to-noise ratio.

The three compounds CaMnBi_2_, SrMnBi_2_ and BaMn_2_Bi_2_ share similar crystal and magnetic structures, as shown in Fig. 1a–c and their Raman spectra exhibit a common feature around 500∼800 cm^−1^ (Fig. 1d). The measured spectral weights shift towards larger wavenumbers following the order of the ionic radii of Ca, Sr and Ba, and the overall spectral feature remains unchanged under different excitation sources (see the inset in Fig. 1d), which indicates that these spectra come from the Raman process rather than a photoluminescence process. A multi-phonon process is also unlikely, because there are no strong phonon excitations above 300 cm^−1^. Moreover, we analysed the two-magnon Raman process in BaMn_2_Bi_2_, using the exchange couplings *SJ*_1_=21.7(1.5) meV, *SJ*_2_=7.85(1.4) meV and *SJ*_c_=1.26(0.02) meV determined by the neutron-scattering measurements^19^. Our calculations (see Supplementary Note 1 for the method of calculation) produced two-magnon excitations that peak around 650 cm^−1^, in good agreement with our experimental observation. This agreement between experiment and theory further demonstrates that the spectral feature around 500–800 cm^−1^ in these Mn-Bi compounds originates from the two-magnon Raman process.

### Evolution of two-magnon peak with temperature

**Figure 2:**
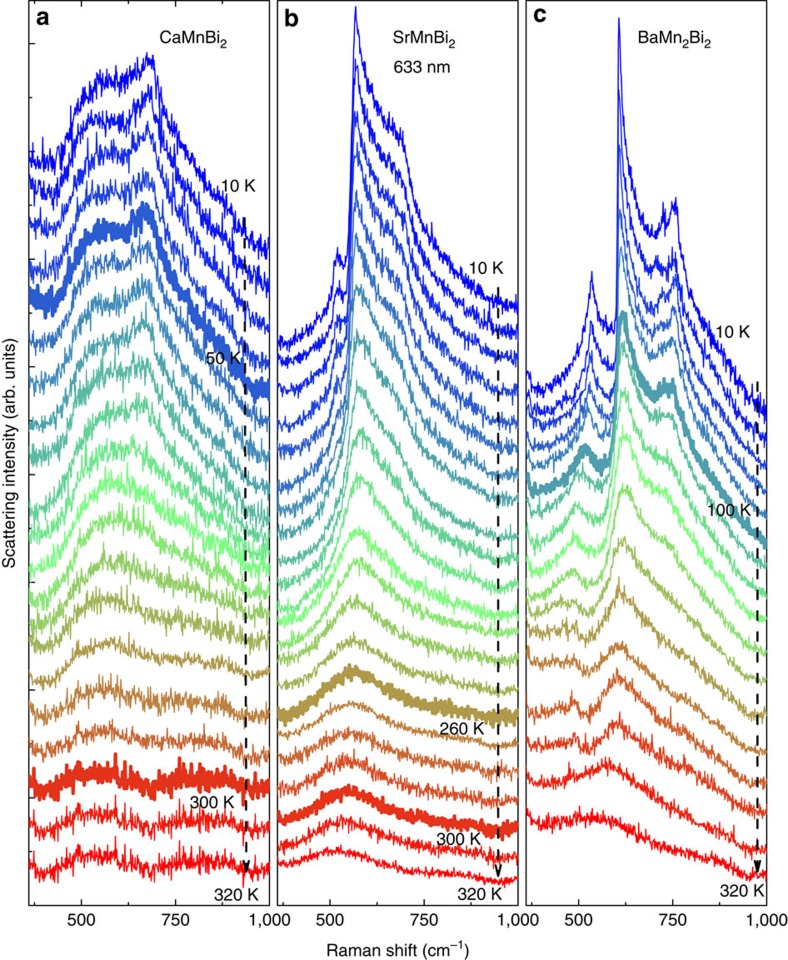
Temperature evolution of the two-magnon spectra. Measured Raman spectra of (**a**) CaMnBi_2_, (**b**) SrMnBi_2_ and (**c**) BaMn_2_Bi_2_. Highlighted are the spectra at 50 and 300 K in **a**, 260 and 300 K in **b**, and at 100 K in **c**, where resistivity and susceptibility anomalies or antiferromagnetic transitions were observed in measurements using other techniques as discussed in the text, but no anomalies are visible in the two-magnon spectra at these temperatures. The spectra were collected in an unpolarized configuration to obtain a better signal-to-noise ratio.

We show in Fig. 2 the evolution of the two-magnon peak with temperature. The spectra from all three compounds exhibit the same trends with increasing temperature, including a shift to lower wavenumbers in peak position, a gradual broadening in peak width and a reduction in intensity, which nevertheless remains visible above the transition temperatures (*T*_N_). These trends are typical for the two-magnon Raman process. The shift of the peak position reflects the energy changes of the large-*q* magnons and magnon–magnon interactions, the peak broadening indicates a decrease of the magnon lifetime and the visibility of the peak structure even above *T*_N_ follows a general feature of the two-magnon process, as the peak is dominated by the magnons at the Brillouin zone boundary where the magnetic correlation remains viable even far above *T*_N_^20^. The peak at ∼500 cm^−1^ is probably associated with a process involving a phonon and a magnon, considering its temperature evolution is similar to that of the two-magnon spectra and there is a strong SOC in the system^21^. The anomalies in resistivity and susceptibility reported at 260 and 50 K in SrMnBi_2_ and CaMnBi_2_, respectively, were attributed to spin realignments or a slight structural change without any anomalies in specific heat^12^,^13^,^14^,^18^. We observed no anomalies in the two-magnon spectra at these temperatures (Fig. 2a,b). Our results thus rule out spin-related processes as the origin of these anomalies, as two-magnon spectra are highly sensitive to variations of the magnetic order in the system.

### Determination of exchange energies

By comparing the characteristic energies extracted from the spectra to the calculated two-magnon density of states, we have determined, for the first time, the exchange energies in SrMnBi_2_ and CaMnBi_2_, as summarized in Table 1 (see Supplementary Notes 2–7, Supplementary Figs 1–3 and Supplementary Tables 1–3 for details). We also have extracted the exchange energies for BaMn_2_Bi_2_ and the values are in good agreement with those determined by the neutron-scattering measurements^19^, which confirms the reliability of the results from the two-magnon Raman spectra. The small difference between the *J*c values extracted by the two methods may have resulted from different sample sources and/or experimental methods.

**Figure 3:**
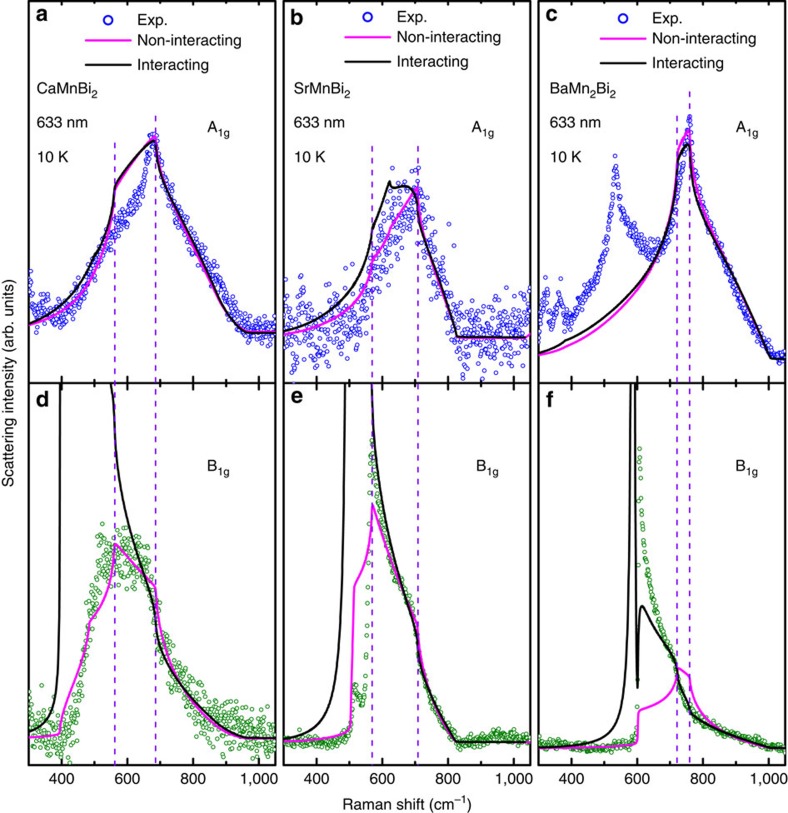
Measured and calculated two-magnon Raman spectra. The low-temperature (10 K) two-magnon Raman spectra in the A_1g_ (**a**–**c**) and B_1g_ (**d**–**f**) channels (circles) compared with the calculated results based on the linear spin–wave theory without considering the magnon–magnon interactions (magenta lines) and the results taking into account the magnon–magnon interactions (black lines). The vertical dashed lines mark the characteristic frequencies that are independent of the symmetries and the magnon–magnon interactions. These two characteristic frequencies, in combination with the cutoff frequencies, determine the exchange energies *J*_1_, *J*_2_ and *J*_c_ (see Supplementary Note 2 and Supplementary Fig. 1 for details).

The very-high-energy resolution of Raman scattering (∼0.1 meV) combined with the sharp two-magnon features observed in all three crystals are expected to produce an accurate determination of the magnetic exchange energy^22^. There is also appropriate verification on the validity of the theoretical model adopted in the present work, which predicts that the characteristic points in two symmetry channels are exactly the same (Fig. 3, A_1g_ and B_1g_, see below). This predicted coincidence is indeed seen in the experimental spectra. There are four available characteristic points in the two-magnon spectra and any three of them can provide the same exchange parameters. We have examined the accuracy of the extracted exchange energies by inputting much larger error bars for the raw data (Supplementary Notes 6 and 7, and Supplementary Figs 2 and 3). In particular for the small *J*_c_, its value is exactly proportional to the width of the plateau between *ω*_1_ and *ω*_2_ (Supplementary Note 2). The value of *J*_c_ read out from Fig. 3 well coincides with those listed in Table 1. It should be emphasized that the same modelling is equally applied to all three systems studied in the present work without any additional constraints. This means that the exchange energies relative to each other are consistently comparable even if their magnitudes may have deviations.

Based on the obtained exchange energies, we have calculated the two-magnon spectra in the A_1g_ and B_1g_ channels (see Supplementary Note 1), where the irreducible representations of the D_4h_ point group, A_1g_ and B_1g_, denote different symmetry channels and can be separated by configuring the polarizations of incident and scattered light. The results (Fig. 3) show that the magnon–magnon interactions have little influence on the A_1g_ spectra but contribute a sharp resonant peak in the B_1g_ channel. Magnon–magnon scattering drives a spectral weight transfer to lower energies and consequently causes such a magnetic exciton-like resonance peak, whose position corresponds to the exciton energy. The simulations including the magnon–magnon interactions produce results in much better agreement with the experimental data for BaMn_2_Bi_2_ compared with the results of the non-interacting calculations. However, the non-interacting results agree better with the experimental spectra in AMnBi_2_. It should be noted that the non-interacting calculations in AMnBi_2_ have some discrepancies with the experimental data in some spectral details. The presence of itinerant electrons, particularly in CaMnBi_2_ and SrMnBi_2_, may be responsible for such deviations. The itinerant electrons tend to reduce the effective intensities of the incident light and contribute to a relatively low signal-to-noise ratio. Furthermore, itinerant electrons also bring higher-order corrections to the linear spin-wave model. Theoretical calculations for such corrections are extremely complicated and have not been archived in the literature; however, such corrections are not expected to affect the main features and characteristic points in the spectra, although they can possibly modify some spectral details.

## Discussions

The strong suppression of the resonant peak in Ca(Sr)MnBi_2_ is associated with the strong SOC effect in the Ca(Sr)Bi layer^16^, which leads to an easy-axis anisotropy (along the *S*^z^ direction) of the exchange couplings, as well as an anisotropic damping of the magnon–magnon interactions that can suppress the resonant peak in the B_1g_ channel of the Raman spectra. Although the resonance stems from magnon–magnon interactions as mentioned above and is strongly suppressed by the SOC effect, the resonance peak intensities may not be a good measure of the interaction or SOC strengths. This is because many basic factors such as distinguished crystal symmetries (the presence of a horizontal mirror plane in SrMnBi_2_ but absent in CaMnBi_2_), magnetic structures (G-type for SrMnBi_2_ but C-type for CaMnBi_2_) and carriers (Dirac electrons dominant in SrMnBi_2_ but Dirac plus ordinary electrons in CaMnBi_2_) are not taken into account. Further insights into this important issue require additional experimental and theoretical investigation.

The in-plane exchange couplings *J*_1_ and *J*_2_ in the three compounds studied here are well correlated with the in-plane lattice constants. SrMnBi_2_ has the largest lattice parameter along its *a* axis and the smallest *J*_1_ and *J*_2_; BaMn_2_Bi_2_ and CaMnBi_2_ have similar *a* values and their *J*_1_ and *J*_2_ are quite close to each other (see Table 1). This close correlation suggests that the in-plane magnetism is well described by the super-exchange mechanism. In sharp contrast, the interlayer coupling *J*_c_ exhibits a highly anomalous behaviour. The distances between the neighbouring MnBi layers in CaMnBi_2_ and SrMnBi_2_ (11.07 Å and 11.57 Å, respectively) are much larger than that in BaMn_2_Bi_2_ (7.34 Å) because of the intercalation of the additional Bi layers in the two magnetic Dirac compounds. At such large interlayer distances, the interlayer coupling *J*_c_ is usually expected to be negligible as suggested by recent neutron measurements and calculations^15^,^18^. In fact, negligible *J*_c_ values have been reported in many other layered compounds with magnetic interlayer distance ≳0.7 nm, such as K_2_NiF_4_, K_2_MnF_4_ and Rb_2_MnF_4_ (refs 23, 24). Surprisingly, the extracted *J*_c_ value for SrMnBi_2_, which has the largest MnBi interlayer distance, is 3.6 times that in BaMn_2_Bi_2_, which has the smallest MnBi interlayer distance. Meanwhile, CaMnBi_2_ also has a larger *J*_c_ ∼1.7 times that of BaMn_2_Bi_2_. This unusual enhancement of *J*_c_ is apparently beyond the standard super-exchange mechanism and indicates novel physics in these magnetic Dirac materials. A key structural feature in the AMnBi_2_ compounds is a Dirac carrier Ca(Sr)Bi layer between the neighbouring magnetic MnBi layers; in contrast, there is only a single layer of Ba^2+^ ions between the neighbouring MnBi layers in BaMn_2_Bi_2_. This structural contrast suggests that the enhanced interlayer magnetic coupling stems from the interplay of magnetism and the Dirac carriers in the intervening Ca(Sr)Bi layer. The spin–fermion systems studied here provide unique insights into the novel physics of the composite magnetic and Dirac electron systems where the layers accommodating itinerant carriers are sandwiched by ordered and insulating magnetic layers. On the other hand, the present material systems do not provide an adequate platform to clearly identify the role of the ordinary electrons. A definitive resolution of this issue requires the synthesis of appropriately structured spin–fermion systems and additional theoretical exploration, which is beyond the scope of our present work.

To understand the interplay of the Dirac carriers and magnetism in SrMnBi_2_ and CaMnBi_2_, especially the enhancement of the coupling *J*_c_ between neighbouring magnetic layers and the modifications of the electronic band structure in the Dirac carrier layers, we consider the following effective spin–fermion model describing both the itinerant electrons in the Bi 6*p*_*x*_ and 6*p*_*y*_ orbits, and interacting local magnetic moments on the Mn ions:


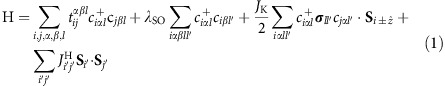


where 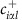 creates an itinerant electron at site *i* in orbit *α* with spin index *l* in the Ca(Sr)Bi Dirac carrier layer, 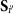 refers to the local moment of the Mn ion below or above the Ca(Sr)Bi layer, 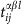 is the hopping integral of the itinerant electrons, *λ*_SO_ is the SOC, *J*_K_ is the Kondo coupling between the itinerant electrons and local moments, and *J*^H^ is the super-exchange coupling between the local moments. Here we use the exchange energies determined from our two-magnon Raman spectra and treat the local moments of the Mn ions as classical spins, which is justified by the large moment of ∼4 μ_B_ per Mn at low temperatures, and the magnetic interaction in the antiferromagnetic (AFM) state is treated in a mean-field approximation.

**Figure 4:**
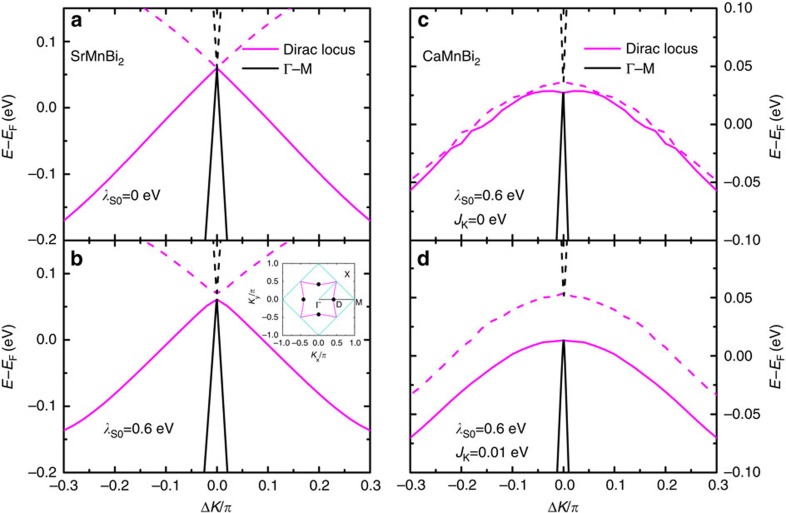
Anisotropic Dirac bands in SrMnBi_2_ and CaMnBi_2_. (**a**,**b**) The dispersion of the Dirac bands (black lines) along the Γ–M direction of the Brilluion zone and the locus of the crossing point energy of the Dirac bands (magenta lines) without or with the SOC *λ*_SO_ for SrMnBi_2_. The lower and upper branches are shown in solid and dashed lines. The inset in **b** shows the isolated Dirac points (black dots D) in SrMnBi_2_ and continuous Dirac points (Dirac locus, magenta line) in CaMnBi_2_. (**c**,**d**) The corresponding Dirac bands (black lines) along the Γ–M direction and the Dirac locus (magenta lines) with the SOC *λ*_SO_ for CaMnBi_2_ at two different values of the exchange coupling *J*_K_. Here, the gap size between the lower and upper Dirac bands are dominated by the exchange coupling *J*_K_ instead of the SOC *λ*_SO_. Here, Δ*k*=|*k*−*k*_D_|, where *k*_D_ is the moment of the Dirac point. The Γ–M direction and Dirac locus direction are along the black line and magenta curve in the inset in **b**, respectively.

The results of our model calculations show that both SrMnBi_2_ and CaMnBi_2_ possess anisotropic Dirac bands, but the processes for the gap opening between the upper and lower Dirac bands are very different in these two compounds. As already noticed in a previous study^15^, the different arrangement of the Ca or Sr cations leads to a gap opening along a general direction in SrMnBi_2_, but not in CaMnBi_2_. As a result, there are four isolated anisotropic Dirac points along the Γ–M direction in SrMnBi_2_, but a line of continuous Dirac points is present in CaMnBi_2_ (see Supplementary Note 8, Supplementary Fig. 4 and Supplementary Table 4). We examine the effects of SOC and magnetic order on the gap opening in the Dirac bands. For SrMnBi_2_, as shown in Fig. 4a,b, the SOC opens a small gap (∼0.01 eV for *λ*_SO_=0.6 eV) at the Dirac band along the Γ–M direction and it slightly enhances the existing gap between the lower and upper Dirac bands. The magnetic order has no net effect on the band structure at the mean-field level, as the influence coming from the upper and lower Mn layers exactly cancel out due to the G-AFM order. For CaMnBi_2_, the effect of SOC is similar, which opens a small gap of ∼0.01 eV between the upper and lower Dirac bands. This gap, however, is much smaller than observed in a recent ARPES measurement, which is ∼0.05–0.1 eV.

Surprisingly, we find that in CaMnBi_2_, the C-AFM order introduces a massive term proportional to the sublattice magnetization, 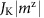, which acts on itinerant electrons, and this term is highly effective in opening a gap in the Dirac bands (Fig. 4c,d). Taking *J*_K_=0.01 eV, the gap increases fivefold to 0.05 eV, which is consistent with the value observed in recent ARPES experiment^16^. Meanwhile, we also estimated the effective *J*_c_ in CaMnBi_2_ driven by the Ruderman-Kittel-Kasuya-Yosida (RKKY) interaction (see Supplementary Note 9). At the same *J*_K_=0.01 eV, we obtained |*J*_c_|∼1 meV, which is in good agreement with the value obtained independently from fitting the Raman spectra (Table 1). These results show consistently that the coupling between the Dirac electrons and local moments has a profound impact on both the effective interlayer magnetic interaction and the Dirac electronic band structure. This finding highlights a powerful characteristic of these magnetic Dirac systems and raises exciting prospects of manipulating these key properties by tuning the interlayer exchange coupling in magnetic Dirac materials. The spin–Fermion model traditionally applies to systems with magnetism and conducting carriers coexisting in the same lattice. The MnBi compounds studied here present a new environment where the magnetic moments and conducting carriers are well separated in different subsystems. This allows an accurate description of the spin–Fermion interaction in these materials and the results offer new insights into the fundamental physics that may inspire innovative design concepts for spintronic applications.

In summary, we have performed a systematic two-magnon Raman study of magnetic Dirac compounds SrMnBi_2_ and CaMnBi_2_. Our measurements combined with model calculations produced, for the first time, an accurate determination of the exchange energies, which allow a quantitative understanding of the novel physics in these materials. A comparison with the reference compound BaMn_2_Bi_2_ reveals that the interlayer exchange couplings are significantly enhanced and the magnon–magnon interactions are suppressed by the Dirac carrier layers. We further investigated the effects of magnetism on the band structure of Dirac carriers and found that the magnetic order has drastic effects on the gap opening in the Dirac bands in CaMnBi_2_, which explains recent ARPES measurements. The discovery of the intriguing interplay of Dirac carriers and magnetism sheds new light on the rich physics in magnetic Dirac materials. Our reported work sets key benchmarks for these distinct systems containing coupled but well-separated magnetic and Dirac carrier layers that can be accurately described by the spin–Fermion model. These results unveil new fundamental physics and pave the way for innovative design and development of magnetic Dirac devices for spintronic applications.

## Methods

### Experimental details

High-quality crystals of BaMn_2_Bi_2_, SrMnBi_2_ and CaMnBi_2_ were grown by self-flux method. The details of crystal growth can be found elsewhere^11^,^14^,^18^,^25^. The AF transition temperatures of SrMnBi_2_ and CaMnBi_2_ could be found in ref. 18, where magnetic susceptibilities and resistivities were measured in the same batch of crystals as used in our measurements. Raman measurements were performed with a Jobin Yvon HR800 single-grating-based micro-Raman system equipped with a volume Bragg grating low-wavenumber suite, a liquid-nitrogen-cooled back-illuminated charge-coupled device detector and a 633 nm laser (Melles Griot). The laser was focused into a spot of ∼5 μm in diameter on sample surface, with a power <100 μW, to avoid overheating.

### Data availability

The data that support the findings of this study are available from the corresponding uthor upon reasonable request.

## Additional information

**How to cite this article:** Zhang, A. *et al*. Interplay of Dirac electrons and magnetism in CaMnBi_2_ and SrMnBi_2_. *Nat. Commun.* **7,** 13833 doi: 10.1038/ncomms13833 (2016).

**Publisher's note:** Springer Nature remains neutral with regard to jurisdictional claims in published maps and institutional affiliations.

## Supplementary Material

Supplementary Information Supplementary Figures, Supplementary Tables, Supplementary Notes and Supplementary References.

## Figures and Tables

**Table 1:** The magnetic exchange energies extracted from the Raman spectra.

	***SJ***_**1**_**(meV)**	***SJ***_**2**_**(meV)**	***SJ***_**c**_**(meV)**	***a*** **(Å)**	***d*** **(Å)**
CaMnBi_2_	20.77 (0.79)	7.29 (0.48)	−1.31 (0.10)	4.50*	11.07*
SrMnBi_2_	16.00 (0.30)	4.75 (0.17)	2.92 (0.09)	4.58*	11.57*
BaMn_2_Bi_2_	21.45 (0.32)/21.7(1.5)†	6.26 (0.20)/7.85(1.4)†	0.78 (0.08)/1.26(0.02)†	4.49†	7.34†

Here, *a* is the in-plane lattice constant and *d* the distance between the neighbouring MnBi layers.

^*^ref. 18.

^†^ref. 19.
