# Supplementary material for: Interplay of Dirac electrons and magnetism in CaMnBi2 and SrMnBi2
Source: Nat Commun. 2016 Dec 16;7:13833. doi: 10.1038/ncomms13833 (PMC5172363; doi:10.1038/ncomms13833)
Supplement: Supplementary Information — Supplementary Figures, Supplementary Tables, Supplementary Notes and Supplementary References. [file ncomms13833-s1.pdf]

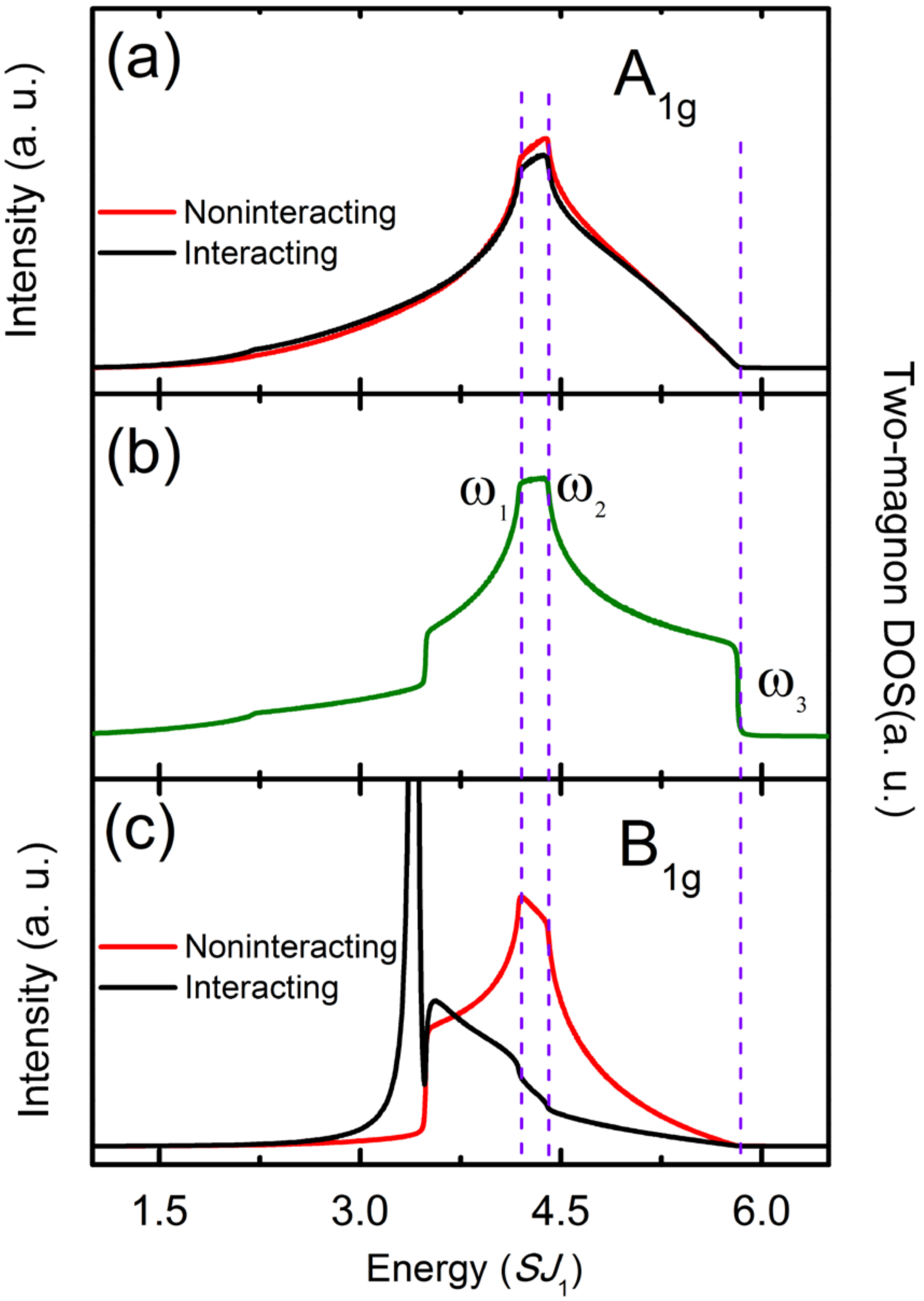

Supplementary Figure 1: **The three characteristic frequencies ω_1, ω_2 and ω_3 in the two-magnon Raman spectra and van-Hove singularities in the two-magnon DOS.**

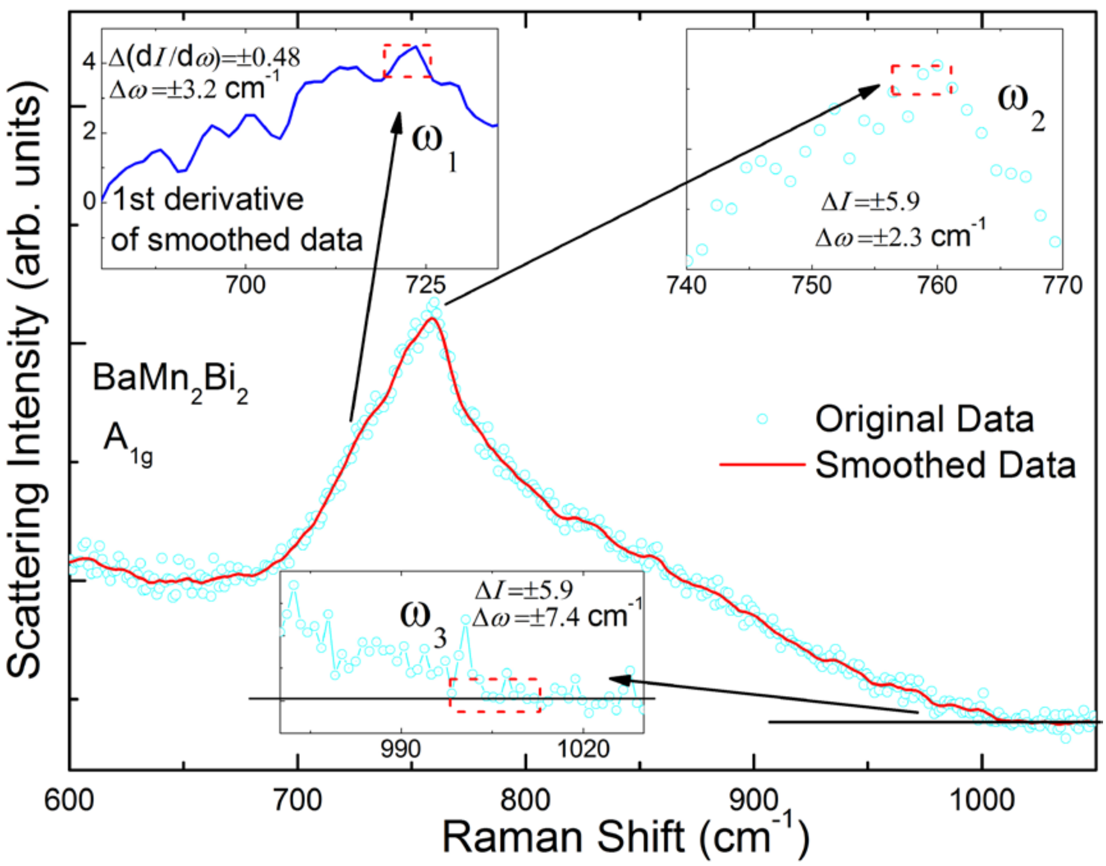

Scattering Intensity (arb. units)
Raman Shift (cm^{-1})
BaMn$_2$Bi$_2$
A$_{1g}$
Original Data
Smoothed Data
Δ(dI/dω)=±0.48
Δω=±3.2 cm^{-1}
1st derivative
of smoothed data
ω$_1$
ω$_2$
ΔI=±5.9
Δω=±2.3 cm^{-1}
ω$_3$
ΔI=±5.9
Δω=±7.4 cm^{-1}

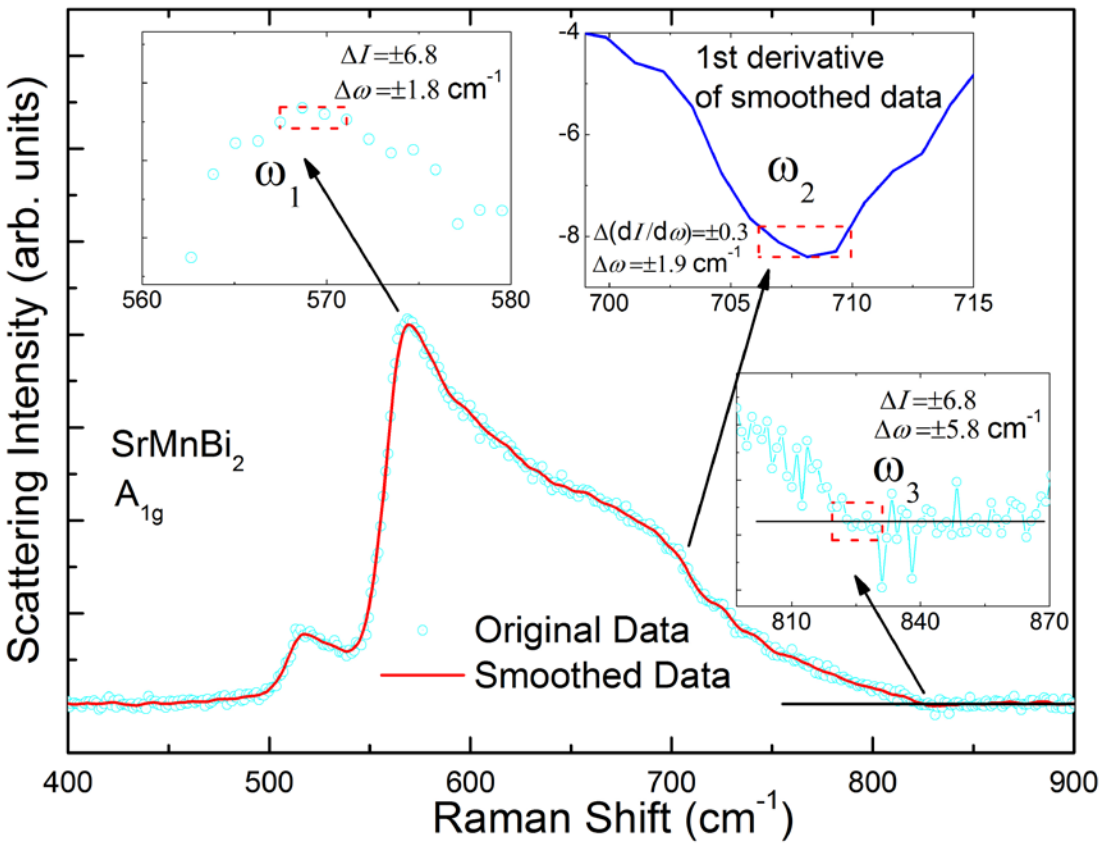

Scattering Intensity (arb. units)
Raman Shift (cm^{-1})
SrMnBi$_2$
A$_{1g}$
Original Data
Smoothed Data
ΔI=±6.8
Δω=±1.8 cm^{-1}
ω$_1$
1st derivative
of smoothed data
ω$_2$
Δ(dI/dω)=±0.3
Δω=±1.9 cm^{-1}
ΔI=±6.8
Δω=±5.8 cm^{-1}
ω$_3$

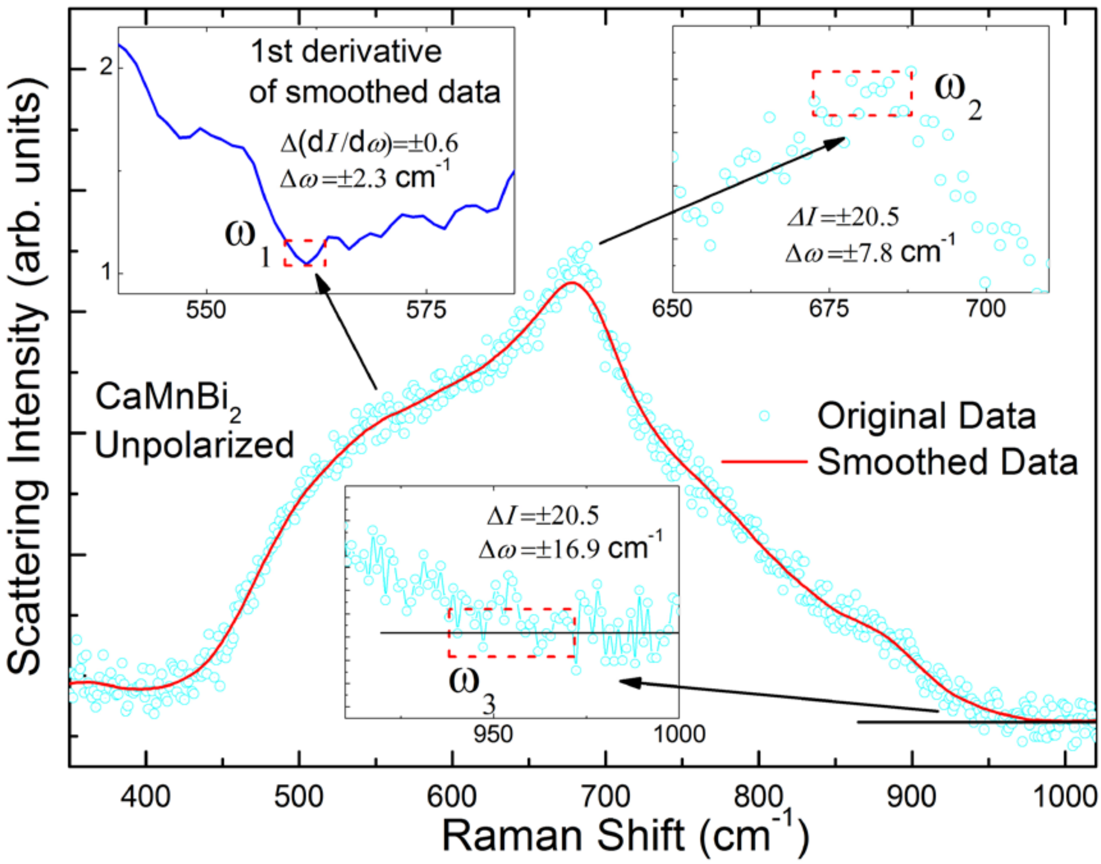

Supplementary Figure 2: Determination of characteristic spectral points in $BaMn_2Bi_2$ (upper), $SrMnBi_2$ (medium) and $CaMnBi_2$ (lower). The red dashed boxes in all the insets indicate the error ranges, as described in the above text. The black lines are the baselines given by fitting the background at the high-frequency end.

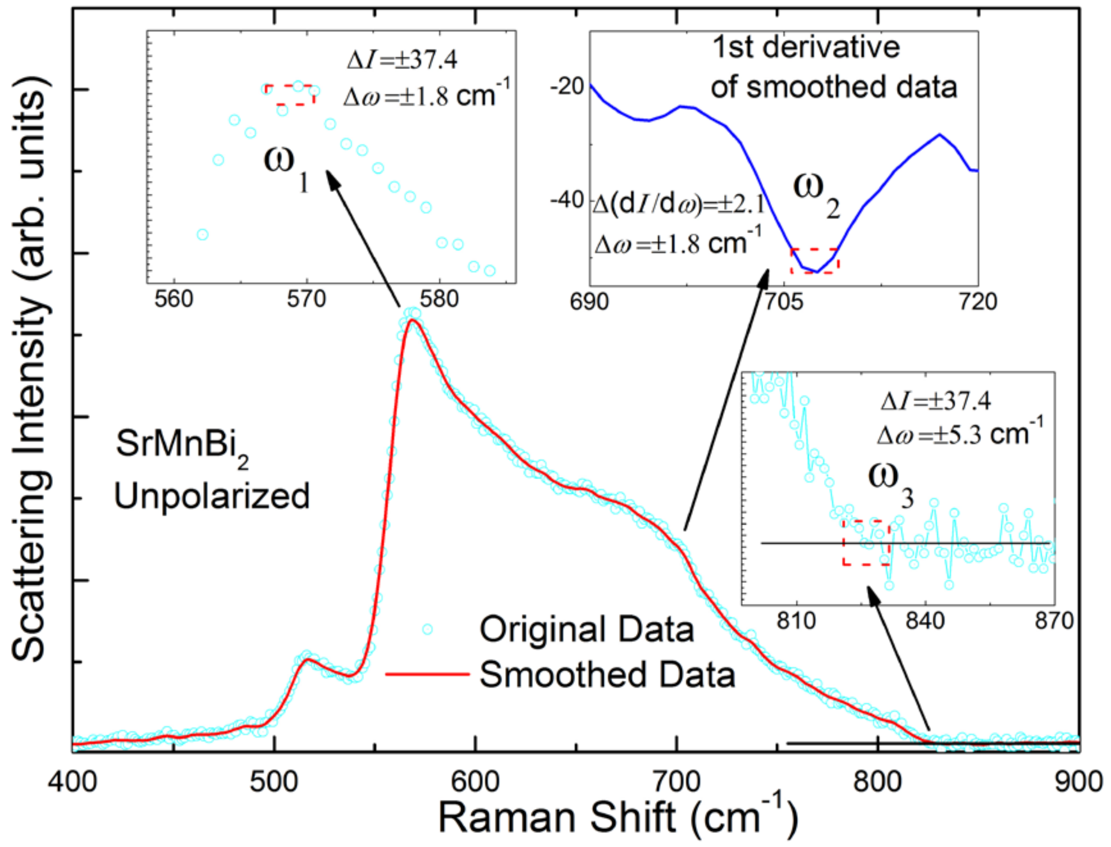

Supplementary Figure 3: Determination of characteristic spectral points in $SrMnBi_2$ using the unpolarized spectra. The red dashed boxes in the insets indicate the error ranges.

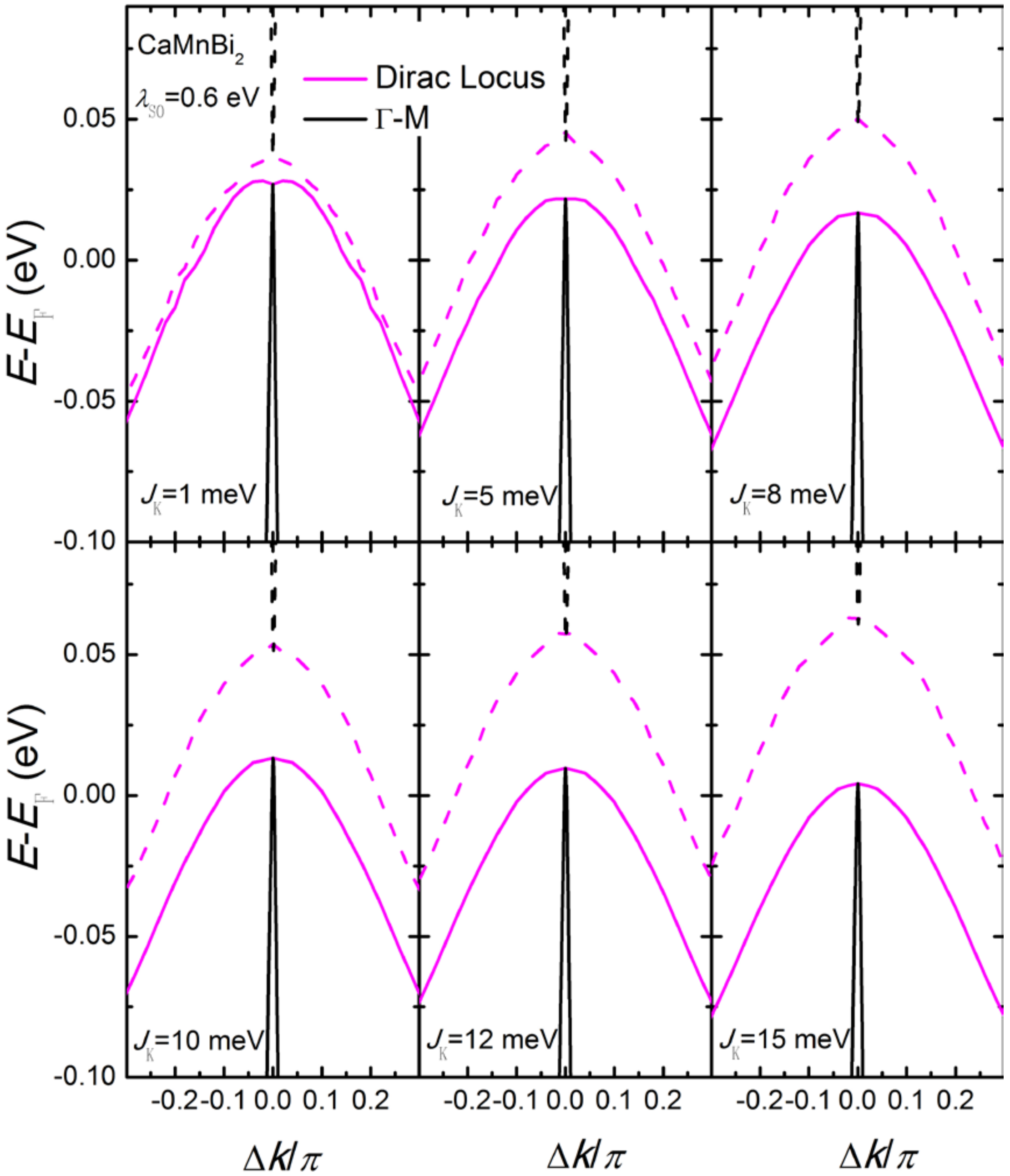

Supplementary Figure 4: The energy gaps of anisotropic Dirac bands with various J_k in $CaMnBi_2$. The figure shows the dispersion of the Dirac bands (black lines) along the Γ-M direction of the Brilluion zone and the locus of the crossing point energy of the Dirac bands (magenta lines) for $CaMnBi_2$. The lower and upper branches are shown in solid and dashed lines.

Supplementary Table 1: Three characteristic frequencies obtained from the experimental spectra and the extracted exchange parameters with the error bars obtained through the error estimation described in Supplementary Note 3.

	$CaMnBi_2$	$SrMnBi_2$	$BaMn_2Bi_2$
ω_1 (cm^{-1})	561.2(2.3)	569.3(1.8)	722.6(3.2)
ω_2 (cm^{-1})	680.2(7.8)	708.1(1.9)	758.7(2.3)
ω_3 (cm^{-1})	954.9(16.9)	825.2(5.8)	1005.5(7.4)
SJ_1 (meV)	20.77(0.79)	16.00(0.30)	21.45(0.32)
SJ_2 (meV)	7.29(0.48)	4.75(0.17)	6.26(0.20)
SJ_c (meV)	-1.31(0.10)	2.92(0.09)	0.78(0.08)

Supplementary Table 2: Comparison of the parameters in $SrMnBi_2$ extracted from the polarized and unpolarized spectra, respectively.

$SrMnBi_2$	Polarized (A_{1g})	Unpolarized
ω_1 (cm^{-1})	569.2(1.8)	568.7(1.8)
ω_2 (cm^{-1})	708.1(1.9)	707.3(1.8)
ω_3 (cm^{-1})	825.2(5.8)	826.2(5.3)
SJ_1 (meV)	16.00(0.30)	16.08(0.27)
SJ_2 (meV)	4.75(0.17)	4.79(0.15)
SJ_c (meV)	2.92(0.09)	2.89(0.08)

Supplementary Table 3: Comparison of exchange interactions for three materials with/without single-ion anisotropy.

		$CaMnBi_2$	$SrMnBi_2$	$BaMn_2Bi_2$
$\frac{D}{J_1} = 0.046$	SJ_1 (meV)	20.85(0.81)	15.97(0.39)	21.24(0.32)
	SJ_2 (meV)	7.76(0.51)	4.97(0.23)	6.51(0.21)
	SJ_c (meV)	-1.23(0.10)	2.70(0.10)	0.72(0.07)
$\frac{D}{J_1} = 0$	SJ_1 (meV)	20.77(0.79)	16.00(0.30)	21.45(0.32)
	SJ_2 (meV)	7.29(0.48)	4.75(0.17)	6.26(0.20)
	SJ_c (meV)	-1.31(0.10)	2.92(0.09)	0.78(0.08)

Supplementary Table 4: The hopping parameters and the chemical potential μ

Model parameters	$SrMnBi_2$ (eV)	$CaMnBi_2$ (eV)
$t^{x}_{1\sigma}$	2.00	2.00
$t^{x}_{1\pi}$	-0.50	-0.50

t_2^{x}	0.12	0.26
μ	0.00	0.11

Supplementary Note 1: Method of calculation for two-magnon Raman scattering spectra

The standard theory of magnetic Raman scattering is based on the Elliott-Fleury-London theory[1]. The Raman scattering operator is given by

$$\hat{O} \sim \sum_{ij} J_{ij} (\hat{\mathbf{e}}_{in} \cdot \hat{\mathbf{d}}_{ij})(\hat{\mathbf{e}}_{out} \cdot \hat{\mathbf{d}}_{ij})\, \mathbf{S}_i \cdot \mathbf{S}_j. \quad (1)$$

Here J_{ij} is the spin exchange interaction between the local moments at site *i* and site *j*, $\hat{\mathbf{e}}_{in}$ and $\hat{\mathbf{e}}_{out}$ are unit polarization vectors of the incoming and scattering light, respectively, and $\hat{\mathbf{d}}_{ij}$ is the vector connecting site *i* and site *j*. The Raman cross section at zero temperature is given by the imaginary part of the correlation function $I(\omega) = -i \int \mathrm{d}t\, e^{i\omega t} \langle \mathcal{T}_t \hat{\mathbf{O}}^{+}(t) \hat{\mathbf{O}}(0) \rangle_0$, where $\langle \ldots \rangle_0$ represents the quantum mechanical average over the ground state, and $\mathcal{T}_t$ is the time ordering operator.

We use the spin-wave approach within the framework of perturbative theory to calculate Raman spectra[2]. By introducing the Holstein-Primakoff transformation, we express spins in A (spin up) and B (spin down) sublattices in terms of H-P boson operators a_i and b_j. The system Hamiltonian $\mathrm{H} = J_1 \sum_{\langle ij \rangle} \mathbf{S}_i \cdot \mathbf{S}_j + J_2 \sum_{\langle\langle ij \rangle\rangle} \mathbf{S}_i \cdot \mathbf{S}_j + J_c \sum_{\langle\langle\langle ij \rangle\rangle\rangle} \mathbf{S}_i \cdot \mathbf{S}_j$ is expanded in powers of $1/S$ as:

$$\mathrm{H} \approx \mathrm{S}^2 \left(E_0 + \frac{1}{\mathrm{S}} \hat{H}_0 + \frac{1}{\mathrm{S}^2} \hat{H}_1 + \cdots \right) \quad (2)$$

Here E_0 is a constant classical energy, $\hat{H}_0$ contains quadratic terms of magnons and represents the linear spin-wave (LSW) correction to the classical energy, and $\hat{H}_1$ contains magnon quartic terms, representing two-body magnon-magnon interactions. The higher order terms are ignored in the present treatment.

To calculate the correlation function $I(\omega)$, we first apply Fourier and Bogoliubov transformations to diagonalize the quadratic LSW part $\hat{H}_0$ in terms of Bogoliubov magnons $\alpha_{\mathbf{k}}$ and $\beta_{-\mathbf{k}}$. The $\hat{H}_1$ part then contains Bogoliubov magnons in quartic order, and the magnon-pair-scattering term $\alpha_{\mathbf{k}}^{+} \beta_{-\mathbf{k}}^{+} \beta_{-\mathbf{k}'} \alpha_{\mathbf{k}'}$ in $\hat{H}_1$ is treated within the ladder approximation. The interaction vertex is reserved to the lowest $(1/\mathrm{S})^0$ order, and the ladder diagrams are summed up exactly.

Supplementary Note 2: Determination of exchange energies J_1, J_2 and J_c

We determine the exchange couplings J_1, J_2 and J_c from three characteristic frequencies ω_1, ω_2 and ω_3, which, as shown in Supplementary Figure 1, correspond to the frequencies of the shoulders and cut-off of the A_{1g} Raman spectra. These frequencies are associated with the van-Hove singularities in the

two-magnon density of states (DOS), and are not shifted by the magnon-magnon interactions within our approximations (Supplementary Figure 1). In the linear spin-wave theory, these frequencies take the following analytical form:

1) G-type AFM

$$\omega_1 = 4S(2J_1 + J_c)\sqrt{\frac{J_1-2J_2}{J_1+2J_2}} \quad (3)$$

$$\omega_2 = \frac{4S[2J_1(J_1-2J_2)+J_c(J_1+2J_2)]}{\sqrt{J_1^2-4J_2^2}} \quad (4)$$

$$\omega_3 \gtrsim 4S\sqrt{\frac{(2J_1-2J_2+J_c)[2(J_1-J_2)(J_1^2-2J_1J_2+2J_2^2)+J_c(J_1-2J_2)^2]}{J_1^2-2J_1J_2+J_2(2J_2-J_c)}} \quad (5)$$

$$\omega_2 - \omega_1 = SJ_c \times \frac{16J_2/J_1}{\sqrt{1-(2J_2/J_1)^2}} \quad (6)$$

which holds when J_c<4J_2 and $J_c < \frac{2J_1(J_1-2J_2)}{J_1+2J_2}$;

2) C-type AFM

$$\omega_1 = 8SJ_1\sqrt{\frac{J_1-2J_2}{J_1+2J_2}} \quad (7)$$

$$\omega_2 = \frac{8SJ_1(J_1-2J_2-J_c)}{\sqrt{J_1^2-4J_2^2}} \quad (8)$$

$$\omega_3 = 8S(J_1 - J_2 - J_c) \quad (9)$$

$$\omega_2 - \omega_1 = |SJ_c| \times \frac{8}{\sqrt{1-(2J_2/J_1)^2}} \quad (10)$$

which holds when $|J_c| < \frac{J_1(J_1-2J_2)}{2J_2}$, and these constraints for both cases are valid in the cases studied in the present work. From the experimentally determined frequencies, we can extract the three exchange couplings J_1, J_2 and J_c using the above formulas.

Supplementary Note 3: Error estimation of the exchange energies

The characteristic frequencies can be expressed as

$$\boldsymbol{\omega} = \boldsymbol{\omega}(\mathbf{J}) \quad (11)$$

The experimentally determined frequencies have errors around their averages

$$\boldsymbol{\omega} = \bar{\boldsymbol{\omega}} + \delta\boldsymbol{\omega} \quad (12)$$

The corresponding exchange energies can then be written as $\mathbf{J} = \bar{\mathbf{J}} + \delta\mathbf{J}$, where $\bar{\mathbf{J}}$ is determined via the equation $\bar{\boldsymbol{\omega}} = \boldsymbol{\omega}(\bar{\mathbf{J}})$, and $\delta\mathbf{J}$ is obtained through the expansion

$$\delta\boldsymbol{\omega} = \left(\frac{\partial\boldsymbol{\omega}}{\partial\mathbf{J}}\right)|_{\mathbf{J}=\bar{\mathbf{J}}}\, \delta\mathbf{J}, \quad (13)$$

where $\left(\frac{\partial \boldsymbol{\omega}}{\partial \mathbf{J}}\right)|_{\mathbf{J}=\bar{\mathbf{J}}}$ is the Jacobi's determinant, which leads to

$$\delta \mathbf{J} = [\left(\frac{\partial \boldsymbol{\omega}}{\partial \mathbf{J}}\right)|_{\mathbf{J}=\bar{\mathbf{J}}}]^{-1}\, \delta \boldsymbol{\omega}. \quad (14)$$

Supplementary Note 4: Effects of possible spin anisotropy on the exchange energies in $SrMnBi_2$ and $CaMnBi_2$

In Sr(Ca)$MnBi_2$ materials Dirac carriers in Ca(Sr)Bi layers are subjected to SOC effect which may introduce spin anisotropy in the exchange couplings between Mn ions. To take into account the spin anisotropy effect we consider the following model

$$\mathrm{H} = J_1 \sum_{\langle ij \rangle}[S_i^z S_j^z + \frac{\eta}{2}(S_i^+ S_j^- + S_i^- S_j^+)] + J_2 \sum_{\langle\langle ij \rangle\rangle} \mathbf{S}_i \cdot \mathbf{S}_j + J_c \sum_{\langle\langle\langle ij \rangle\rangle\rangle} \mathbf{S}_i \cdot \mathbf{S}_j, \quad (15)$$

where η is the parameter describing the anisotropy, and $\eta < 1$ ensures that spins are aligned along the z direction.

To determine the parameters J_1, J_2, J_c and η, we make use of an additional characteristic point in the measured Raman spectra, namely the absorption edge in the B_{1g} channel. Following the same procedure described above, we find that compared to the isotropic model the difference in the exchanges energies caused by the anisotropy is less than 10%, which indicates that the results obtained using the isotropic model are quite reasonable.

Supplementary Note 5: Effects of single-ion anisotropy in $CaMnBi_2$, $SrMnBi_2$ and $BaMn_2Bi_2$

It has been reported in inelastic neutron scattering experiments that in $BaMn_2Bi_2$ materials there is a spin gap of 16 meV[4]. The magnetic excitations can be well fit to a Heisenberg model plus single-ion anisotropy terms[4]

$$\mathrm{H} = \sum_{ij} J_{ij} \mathbf{S}_i \cdot \mathbf{S}_j - D \sum_i (S_i^z)^2, \quad (31)$$

where $D > 0$ ensures that spins are aligned along the z direction.
We find that all characteristic frequencies will be shifted in presence of finite D terms. However, the relation

$$\omega_2 - \omega_1 = SJ_c \times \frac{16 J_2/J_1}{\sqrt{1-(2J_2/J_1)^2}} \quad (32)$$

for G-type AFM and

$$\omega_2 - \omega_1 = |SJ_c| \times \frac{8}{\sqrt{1-(2J_2/J_1)^2}} \quad (33)$$

for C-type AFM still remains unchanged. This implies that the width of the shoulders in A_{1g} spectra still has no direct relations with D terms. Using the value $D/J_1 = 0.046$ extracted in INS experiments for $BaMn_2Bi_2$ materials[4], we can calculate the exchange interactions as is shown in Supplementary Table 3.

We can see that in presence of D terms, the exchange interactions are slightly changed. The interchange coupling SJ_c becomes slightly smaller in presence of D, but the difference is less than 10%. It is not surprising that D terms can generate a relatively big spin gap but do not strongly affect the exchange interaction results extracted from Raman experiments, since D terms mainly affect low energy magnetic excitations, but Raman techniques mainly probe high energy physics.

Supplementary Note 6: Determination of characteristic frequencies and their errors

The spectra for $BaMn_2Bi_2$ and $SrMnBi_2$ have a good signal-to-noise level, and the frequencies can be directly read out from the raw spectra (ω_2 in $BaMn_2Bi_2$ and ω_1 in $SrMnBi_2$) or obtained from taking the first derivative (ω_1 in $BaMn_2Bi_2$ and ω_2 in $SrMnBi_2$). For $CaMnBi_2$, one must be more careful as the original polarized spectra have a higher noise level, making it hard to accurately determine the characteristic spectral points. On the other hand, spin-wave calculations indicate that both polarized spectra (A_{1g} and B_{1g}) have exactly the same characteristic points. This means that the combined A_{1g} and B_{1g} spectra, i.e., the unpolarized data, possess the same characteristic spectral points. This enables an alternative scenario, where we have collected many unpolarized spectra at 10 K with a much better signal-to-noise ratio, as shown in Supplementary Figure 2. Using the unpolarized spectra (A_{1g}+B_{1g}), one can easily determine the characteristic frequencies and their errors, in the same way as done above for $BaMn_2Bi_2$ and $SrMnBi_2$. We have examined the validity of the method using $SrMnBi_2$, in which there is little difference between the parameters derived from the polarized spectra and the unpolarized ones, respectively (Supplementary Note 7).

Then we can extract the frequency parameters and quantitatively estimate the associated errors in the following procedure (see Supplementary Figure 2). 1) A general linear fitting was made for a linear region selected from the raw spectra or the first-derived data. This gives the standard deviations in intensity (or its derivative); 2) For ω_1 and ω_2 associated with the local maximums/minimums of the raw spectra or the first-derivatives, we can identify a region which starts from the maximums/minimums and vertically extends by double standard deviations (the heights of the red dashed error boxes). All the data points in the region are possible maximums/minimums. This simply fixes the corresponding deviations in frequency (the widths of the error boxes) and provides the standard errors; 3) For ω_3 not associated with a local extreme, we first determined the baselines (black lines) by fitting the background at the

high-frequency end. The left bound of the error box is reached when the positive intensity deviations from a baseline begin to exceed the standard deviations estimated in the first step. Similarly the right bound is defined at the position where the intensity deviations approach the standard ones. This gives the frequency parameter ω_3 and the associated errors. The characteristic frequencies obtained from the experimental spectra strictly following the above procedure and the extracted exchange parameters with the error bars, are listed in Supplementary Table 1.

Supplementary Note 7: Comparison of the parameters extracted from the polarized and unpolarized spectra in $SrMnBi_2$

Following the procedure described in Supplementary Note 6, we can also obtain the frequency parameters in $SrMnBi_2$ with the unpolarized spectra (see Supplementary Figure 3). And the obtained characteristic frequencies and the extracted exchange parameters from the polarized and unpolarized spectra, are listed in Supplementary Table 2 for comparison. There is little difference between both cases. This demonstrates that the unpolarized spectra work well as the polarized ones in obtaining the characteristic frequency points.

Supplementary Note 8: Electronic band structures and Dirac points in $SrMnBi_2$ and $CaMnBi_2$

We consider the spin-fermion Hamiltonian introduced in the main text

$$\mathrm{H} = \sum_{\mathrm{i,j},\alpha,\beta,\mathrm{l}} t_{\mathrm{ij}}^{\alpha\beta\mathrm{l}} \mathrm{c}_{\mathrm{i}\alpha\mathrm{l}}^{+} \mathrm{c}_{\mathrm{j}\beta\mathrm{l}} + \lambda_{\mathrm{SO}} \sum_{\mathrm{i}\alpha\beta\mathrm{l}\mathrm{l}'} \mathrm{c}_{\mathrm{i}\alpha\mathrm{l}}^{+} \mathrm{c}_{\mathrm{i}\beta\mathrm{l}'} + \frac{J_{\mathrm{K}}}{2} \sum_{\mathrm{i}\alpha\mathrm{l}\mathrm{l}'} \mathrm{c}_{\mathrm{i}\alpha\mathrm{l}}^{+} \boldsymbol{\sigma}_{\mathrm{l}\mathrm{l}'} \mathrm{c}_{\mathrm{j}\alpha\mathrm{l}'} \cdot \mathbf{S}_{\mathrm{i}\pm\hat{\mathrm{z}}} + \sum_{\mathrm{i}'\mathrm{j}'} J_{\mathrm{i}'\mathrm{j}'}^{\mathrm{H}} \mathbf{S}_{\mathrm{i}'} \cdot \mathbf{S}_{\mathrm{j}'} \quad (16)$$

The first term is a two-orbital tight-binding model for the itinerant electrons in Bi $6p_x$ and $6p_y$ orbits (in the Sr(Ca)Bi layer). The second term contains the atomic spin-orbit coupling. $\mathbf{S}_{\mathrm{i}\pm\hat{\mathrm{z}}}$ refers to the local moment of Mn in layers above or below the Bi site i. For simplicity, we do not consider the influence of the Bi $6p_z$ orbit. Since the observed magnetic moments in these materials are about 4 μ_B per Mn, we treat $\mathbf{S}_{\mathrm{i}'}$ as classical spins. We take the $J_{\mathrm{i}'\mathrm{j}'}^{\mathrm{H}}$ values obtained from the Raman measurements, so that the ground state of the model has either a G-AFM ($SrMnBi_2$) or a C-AFM ($CaMnBi_2$) order. We then treat the effects of the AFM order on the band structure of the itinerant electrons at the mean-field level.

Within above approximations, the Mn local moments serve as local magnetic fields that couple to the itinerant electrons and modify their dispersion. The Hamiltonian is then written as

$$\mathrm{H} \approx H_{\mathrm{TB}} + H_{\mathrm{S}}, \quad (17)$$

where

$$H_{\rm TB} = \sum_{\rm ij\alpha\beta l} t_{\rm ij}^{\alpha\beta l} c_{\rm i\alpha l}^{+} c_{\rm j\beta l} + \lambda_{\rm SO} \sum_{\rm i\alpha\beta ll'} c_{\rm i\alpha l}^{+} c_{\rm i\beta l'}, \tag{18}$$

$$H_{\rm S} = \frac{J_K}{2} \sum_{n\alpha} m_n (c_{n\alpha\uparrow}^{+} c_{n\alpha\uparrow} - c_{n\alpha\downarrow}^{+} c_{n\alpha\downarrow}), \tag{19}$$

and $m_n = \langle S_n^z \rangle$ is the sublattice magnetic moment for n = A, B sublattice. For $SrMnBi_2$, the magnetic order is G-AFM, where the Mn ions in upper and lower layers belong to the A and B sublattices, respectively, and at the mean-field level, the effect from the two layers cancels out exactly, hence H $\approx H_{\rm TB}$. But for $CaMnBi_2$, the C-AFM order induces an uncompensated magnetic field, which acts as a mass term since it has different signs on the two sublattices.

We rewrite $H_{\rm TB}$ into a matrix form[3]:

$$H_{\rm TB} = \begin{pmatrix} H_{AA} + H_{so} & H_{AB} \\ H_{BA} & H_{BB} + H_{so} \end{pmatrix}, \tag{20}$$

where H_{so} and $H_{nn'}$ are 2×2 matrices. The spin-orbit coupling takes the form

$$H_{so} = \begin{pmatrix} 0 & -i\lambda_{so} \\ i\lambda_{so} & 0 \end{pmatrix}, \tag{21}$$

where λ_{so} is the coupling constant. In our calculations, we consider two cases, $\lambda_{so} = 0$, and $\lambda_{so} = 0.6$ eV.[3]

For the hopping integrals, we assume that the dominant terms are the intraorbital ones, and we neglect the interorbital hopping. We then obtain the following hopping matrices for $SrMnBi_2$ and $CaMnBi_2$:

$$H_{AA}^{Sr} = \begin{pmatrix} 2t_2^{xs}\cos(k_x + k_y) - \mu & 0 \\ 0 & 2t_2^{xs}\cos(k_x + k_y) - \mu \end{pmatrix}, \tag{22}$$

$$H_{BB}^{Sr} = \begin{pmatrix} 2t_2^{xs}\cos(k_x - k_y) - \mu & 0 \\ 0 & 2t_2^{xs}\cos(k_x - k_y) - \mu \end{pmatrix}, \tag{23}$$

$$H_{AB}^{Sr} = H_{BA}^{Sr} = \begin{pmatrix} 2(t_{1\sigma}^{xs}\cos k_x + t_{1\pi}^{xs}\cos k_y) & 0 \\ 0 & 2(t_{1\sigma}^{xs}\cos k_y + t_{1\pi}^{xs}\cos k_x) \end{pmatrix}, \tag{24}$$

$$H_{AA}^{Ca} = H_{BB}^{Ca} = \begin{pmatrix} 4t_2^{xc}\cos k_x \cos k_y - \mu & 0 \\ 0 & 4t_2^{xc}\cos k_x \cos k_y - \mu \end{pmatrix}, \tag{25}$$

$$H_{AB}^{Ca} = H_{BA}^{Ca} = \begin{pmatrix} 2(t_{1\sigma}^{xc}\cos k_x + t_{1\pi}^{xc}\cos k_y) & 0 \\ 0 & 2(t_{1\sigma}^{xc}\cos k_y + t_{1\pi}^{xc}\cos k_x) \end{pmatrix}. \tag{26}$$

Here the hopping parameters and the chemical potential μ are determined by fitting to the DFT band structure, and their values are summarized in Supplementary Table 4. Note that due to the buckling of the Sr cations, $H_{AA}^{Sr} \neq H_{BB}^{Sr}$ for general **k**. The band structure can be obtained by diagonalizing the mean-field Hamiltonian. Without the spin-orbit coupling, for $SrMnBi_2$, the energy in each band reads

$E_{1(2),\pm}^{Sr} =$

$$2t_2^{xs}\cos k_x\cos k_y \pm \sqrt{4t_2^{xs}\sin^2 k_x \sin^2 k_y + 4\left(t_{1\sigma(\pi)}^{xs}\cos k_x + t_{1\pi(\sigma)}^{xs}\cos k_y\right)^2} \quad (27)$$

By requiring

$\sqrt{4t_2^{xs}\sin^2 k_x \sin^2 k_y + 4\left(t_{1\sigma(\pi)}^{xs}\cos k_x + t_{1\pi(\sigma)}^{xs}\cos k_y\right)^2} = 0$, we obtain four Dirac points. For $t_{1\sigma}^{xs} > t_{1\pi}^{xs}$, they are located at $k_x = 0$, $k_y = \pm\text{arccos}\,(-t_{1\pi}^{xs}/t_{1\sigma}^{xs})$, and $k_y = 0$, $k_x = \pm\text{arccos}\,(-t_{1\pi}^{xs}/t_{1\sigma}^{xs})$. Focusing on one Dirac point $k_y = 0$, $k_x = \pm\text{arccos}\,(-t_{1\pi}^{xs}/t_{1\sigma}^{xs})$, the dispersion is very anisotropic, as shown in Fig. 4(a) in the main text. Turning on the spin-orbit coupling pushes apart the Dirac points as shown in Fig. 4(b).

Following the same procedure, we obtain the electronic band structure for $CaMnBi_2$. Without the spin-orbit coupling,

$$E_{1(2),\pm}^{Ca} = 2t_2^{xc}\cos k_x \cos k_y \pm \sqrt{\tilde{J}_k^2 + 4\left(t_{1\sigma(\pi)}^{xc}\cos k_x + t_{1\pi(\sigma)}^{xc}\cos k_y\right)^2} \quad (28)$$

where $\tilde{J}_k = J_k|m_n|$ is the effective magnetic field to itinerant electrons due to the C-AFM order. If we neglect the magnetic coupling, the band structure has continuous Dirac points along the lines $k_x = \pm\text{arccos}(-t_{1\pi}^{xc}/t_{1\sigma}^{xc}\cos k_y)$ and $k_y = \pm\text{arccos}(-t_{1\pi}^{xc}/t_{1\sigma}^{xc}\cos k_x)$. The magnetic field term $\tilde{J}_k$ acts as a mass term and opens a gap between the upper and lower branches of the Dirac bands. Note that a finite spin-orbit coupling may also opens a gap, but it is much smaller compared to the one associated with the AFM order (see Fig.4(c)-(d), Supplementary Figure 4).

Supplementary Note 9: Estimate of the RKKY interaction

In the above spin-fermion model, the coupling between itinerant electrons and local moments mediates an RKKY interaction among the local moments:

$$H_{RKKY}(\mathbf{R}_i - \mathbf{R}_j) = \frac{1}{2}J(\mathbf{R}_i - \mathbf{R}_j)\mathbf{S}_i \cdot \mathbf{S}_j, \quad (29)$$

with the RKKY coupling given by

$$J(\mathbf{R}) = 2\left(\frac{J_K}{N}\right)^2 \sum_{\mathbf{k},\mathbf{k}'} \frac{\Theta(E_\mathbf{k}) - \Theta(E_{\mathbf{k}'})}{E_\mathbf{k} - E_{\mathbf{k}'}} e^{i(\mathbf{k}-\mathbf{k}')\cdot\mathbf{R}}, \quad (30)$$

where $J(\mathbf{R}) = J(\mathbf{R}_i - \mathbf{R}_j)$, $\Theta(E_\mathbf{k})$ is the Fermi distribution function. A simple estimate based on the perturbation theory gives the induced RKKY coupling at the order of $(J_K)^2/E_F$, where E_F is the Fermi energy of the relevant conduction band. Taking the value of J_K~10-20 meV, which is estimated from the size of the gap between the two Dirac bands, and E_F~0.1 eV from the fitting to the DFT results, we get that the RKKY-induced interlayer coupling $|J(\hat{z})|$~1-4 meV. These Jc values are compatible with those obtained from our Raman measurements (see Table I in the main text). This agreement with the experimental results suggests the model used in our analysis is valid in understanding the

fundamental mechanisms underlying the novel phenomena observed in our measured Raman spectra. A more accurate quantitative description of the size and sign of the RKKY interaction would require more sophisticated band-structure calculations, which are beyond the scope of the present work.

Supplementary References

1. Fleury, P. A. & Loudon, R. Scattering of light by one- and two-magnon excitations. *Phys. Rev.* **166,** 514-530 (1968).
2. Luo, C., Datta, T. & Yao, D. X. Spectrum splitting of bimagnon excitations in a spatially frustrated Heisenberg antiferromagnet revealed by resonant inelastic x-ray scattering. *Phys. Rev. B* **89,** 165103 (2014).
3. Lee, G., Farhan, M. A., Kim, J. S. & Shim, J. H. Anisotropic Dirac electronic structures of $AMnBi_2$ (A=Sr, Ca). *Phys. Rev. B* **87,** 245104 (2013).
4. Calder, S. *et al.* Magnetic structure and spin excitations in $BaMn_2Bi_2$. *Phys. Rev. B* **89,** 064417 (2014).
